# Canadian French translation of the BREAST-Q Breast Conserving Therapy module

**DOI:** 10.1186/s41687-026-01049-6

**Published:** 2026-03-31

**Authors:** Bahar Rafinejad-Farahani, Luca C. Bernardini, Jean-Marc Bourque, C. Anne Koch, Danielle Rodin

**Affiliations:** 1 Global Cancer Program, Princess Margaret Cancer Centre, Toronto, Canada; 2 Division of Radiation Oncology, Centre Hospitalier de l’Université de Montréal, Montreal, Canada; 3 Department of Social and Preventive Medicine, Centre Hospitalier de l’Université de Montréal, Montreal, Canada; 4 Radiation Medicine Program, Princess Margaret Cancer Centre, Toronto, Canada; 5 Department of Radiation Oncology, University of Toronto, Toronto, Canada

**Keywords:** BREAST-Q, Patient reported outcomes, Translation, Cognitive interviews, Breast-conserving therapy

## Abstract

**Background:**

The BREAST-Q is a widely validated patient-reported outcome measure following breast surgery and adjuvant therapies. Although several language adaptations exist, the Breast Conserving Therapy (BCT) module has not yet been translated into Canadian French. Given that French is an official language in Canada, a Canadian French adaptation is important to enable equitable participation in research. This study aimed to develop a linguistically accurate and culturally appropriate Canadian French translation of two scales from the BCT module.

**Methods:**

Two BREAST-Q BCT module scales (“Satisfaction with Breasts (Postoperative)” and “Adverse Effects of Radiation (Postoperative)”) were translated into Canadian French following established guidelines. The process included two independent forward translations, back translation, back translation review, and cognitive debriefing interviews with five breast cancer patients.

**Results:**

Forward translation identified three English words or phrases not amenable to direct translation, which were resolved by consensus prior to back translation. Cognitive debriefing interviews with breast cancer patients identified eight terms or expressions for refinement; five were incorporated into the final version based on majority patient feedback, including replacement of “tumorectomie” with “lumpectomie” to reflect terminology more familiar to patients.

**Conclusions:**

The Canadian French translation of the BREAST-Q BCT scales provides a linguistically and culturally adapted tool for assessing patient-reported outcomes in breast cancer care. Inclusion of patient feedback ensured clarity and relevance, supporting equitable participation in research and clinical outcome measurement.

## Background

Breast cancer is the most commonly diagnosed cancer among women in Canada [1]. Advances in early detection and breast cancer treatment have contributed to improved survival after diagnosis and recent data has shown that the prevalence of breast cancer survivors in Canada has doubled from 2007 to 2022 [2]. The growing number of breast cancer survivors has heightened attention to the physical, psychological, and social consequences of the disease and its treatment. This has led to a growing emphasis on understanding and optimizing survivors’ health-related quality of life (HRQoL) following treatment.

Patient-reported outcome measures (PROMs) evaluate the impact of healthcare interventions on HRQoL by measuring symptom burden directly from the patient without clinician interpretation [3]. Integration of PROMs into cancer care has contributed to improved HRQoL and survival through more robust measurement of treatment-related toxicity and symptom management [3, 4]. Within breast cancer, the BREAST-Q is an internationally validated instrument for measuring patient-reported outcomes following breast surgery and other adjuvant therapies [5, 6]. Since its development in 2009, it has been widely adopted in both research and clinical practice, and is included in the International Consortium for Health Outcomes Measurement (ICHOM) Standard Set for Breast Cancer [7].

The BREAST-Q is comprised of six procedure-specific modules, one of which is focused on women who have undergone breast conserving therapy (BCT), which typically includes breast-conserving surgery (lumpectomy) and adjuvant radiation. This BCT module is comprised of 12 scales, each measuring an aspect of well-being or satisfaction [8]. The scales span domains including psychosocial, physical, and sexual health, in addition to satisfaction with patients’ breasts and the care received. This module has undergone translation and validation in multiple languages, but it has not yet been adapted for Canadian French. In Canada, where French is one of the two official languages, the availability of a Canadian French version is important for equitable participation in research.

To ensure the BREAST-Q is accessible and culturally applicable in diverse patient populations in Canada, high-quality linguistic translations are required. The objective of this study is to translate two of the BCT module scales (“Satisfaction with Breasts (Postoperative)” and “Adverse Effects of Radiation (Postoperative)”) from version 2.0 of the BREAST-Q BCT Module into Canadian French. This work was conducted as part of a series of translations for the PRESERVE clinical trial (NCT05592938), an international phase II study of repeat breast-conserving surgery followed by partial breast reirradiation as treatment for local recurrence or new primary in the previously irradiated breast.

## Methods

### Ethical approval and consent to participate

The University Health Network Research Ethics Board was consulted, and this study was deemed exempt from formal review. Participants were informed about the purpose of the study and provided verbal agreement to participate.

### Participants and procedures

**Fig. 1:**
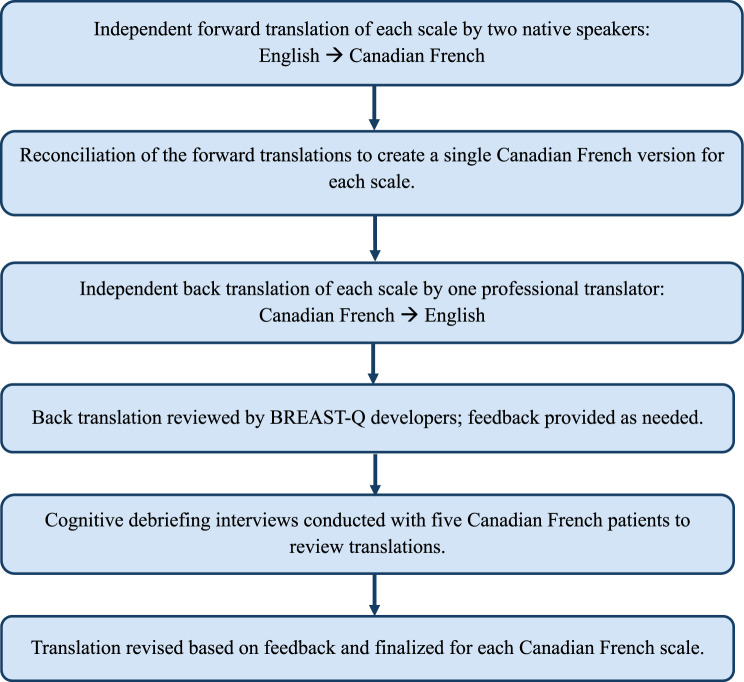
Canadian French translation process for the adaptation of two BREAST-Q Breast Conserving Therapy module scales

Two BREAST-Q BCT module (version 2.0) scales— “Satisfaction with Breasts (Postoperative)” and “Adverse Effects of Radiation (Postoperative)”—were translated into Canadian French. These scales were selected based on their relevance to the study population and treatment-related experiences being evaluated in the PRESERVE clinical trial. The “Satisfaction with Breasts (Postoperative)” scale is an 11-item questionnaire, with four response options ranging from “very dissatisfied” to “very satisfied”. The “Adverse Effects of Radiation (Postoperative)” scale is a six-item questionnaire, with three response options ranging from “a little” to “a lot”. The translation process used a forward–backward translation methodology with reconciliation, followed by semi-structured cognitive interviews with five breast cancer patients to evaluate the translated scales (Fig. 1). The inclusion of five participants is consistent with prior BREAST-Q translation studies and meets the BREAST-Q translation requirement of a minimum of five patients for cognitive debriefing interview [9–11].

A license to use the BREAST-Q was obtained from the copyright holders (McMaster University, Memorial Sloan Kettering Cancer Center, and Mass General Brigham), and permission was granted by the BREAST-Q developers to translate the two scales into Canadian French, in accordance with their guidelines (Fig. 1). This approach is consistent with the International Professional Society for Health Economics and Outcomes Research (ISPOR). Data sharing is not applicable to this study as no datasets were generated or analysed.

### Translations

Two native Canadian French speaking clinical research staff at the Centre hospitalier de l’Université de Montréal (University of Montréal Health Centre) independently translated the scales from English to Canadian French (forward translation). The two translations were compared by these forward translators and reconciled into a single version for each scale. These reconciled versions were then independently back translated into English by a professional translator contracted through the University Health Network’s Interpretation and Translation Services, who was blinded to the original English scales to mitigate bias.

The back-translation was reviewed by the BREAST-Q developers for conceptual equivalence across instructions, response options, or question items. Any discrepancies were addressed through re-translation. Revised versions were resubmitted for final approval prior to the subsequent cognitive interviews.

### Cognitive interviews

Semi-structured cognitive interviews were conducted via Zoom with five female individuals who self-identified as having a history of breast cancer treatment, which included radiotherapy, and who were residing in Canada and fluent in Canadian French.

The cognitive interviews assessed the clarity and comprehensibility of the translated scales. If participants expressed confusion or uncertainty regarding the instructions, response options, or question items, interviewers clarified the intended meaning and invited suggestions for improvement. Revisions were made to any content identified as problematic by at least three participants, following practices for cognitive debriefing in instrument validation [9]. Transcripts of the cognitive interviews, along with interviewer notes, were reviewed and synthesized to inform final revisions. The finalized Canadian French versions of both scales were submitted to the BREAST-Q developers for approval prior to future distribution and use.

## Results

The forward-back translation process identified minor challenges related to the linguistic and cultural adaptation of the two scales. During the forward translation stage, translators identified three English terms or phrases in the two scales that lacked direct equivalents in Canadian French. For instance, the word “breast” and certain response options, such as “somewhat satisfied,” required consideration, as multiple Canadian French translations were possible depending on the intended nuance and context. Furthermore, the question item “how your lumpectomy breast sits/hangs” presented a challenge, as no natural Canadian French expression adequately conveyed the intended anatomical meaning. These issues were resolved through consensus between the two forward translators. The subsequent back translation did not reveal any major discrepancies.

Cognitive debriefing interviews were conducted with five female breast cancer patients who were fluent in both English and Canadian French. Each participant individually reviewed both translated scales and reported no difficulties with the instructions, response options, or items. However, participants identified several terms or phrases for refinement in grammar, wording, or phrasing. In total, eight items were identified: six from the “Satisfaction with Breasts (Postoperative)” scale, and two from the “Adverse Effects of Radiation (Postoperative)” scale. Of these, five lead to a modification in the final translated version (Table 1). For example, the term “tumorectomie” was revised to “lumpectomie” to align with terminology used in Canadian French clinical settings.

**Table 1:** Translation revisions identified during cognitive debriefing in two scales of BREAST-Q Breast Conserving Therapy module

Original English Item	Initial Canadian French Translation	Number of Feedback Responses	Feedback Summary	Final Canadian French Translation
**“Satisfaction with Breasts (Postoperative)” scale**				
The following questions are about your breasts and your breast cancer treatment (by treatment, we mean lumpectomy with or without radiation). If you have had a lumpectomy and radiation of both breasts, answer these questions thinking of the breast you are least satisfied with. With your breasts in mind, in the past week, how satisfied or dissatisfied have you been with:	Les questions suivantes concernent vos seins et votre traitement contre le cancer du sein (par traitement, nous entendons une tumorectomie avec ou sans radiothérapie). Si vous avez subi une tumorectomie et une radiothérapie des deux seins, répondez à ces questions en pensant au sein dont vous êtes le moins satisfait. En gardant à l’esprit vos seins, au cours de la semaine dernière, dans quelle mesure avez-vous été satisfait(e) ou insatisfait(e):	5	”Lumpectomie” is a preferable word to “tumorectomie” to describe a lumpectomy.	Les questions suivantes concernent vos seins et votre traitement contre le cancer du sein (par traitement, nous entendons une lumpectomie avec ou sans radiothérapie). Si vous avez subi une lumpectomie et une radiothérapie des deux seins, répondez à ces questions en pensant au sein dont vous êtes le moins satisfait. En gardant à l’esprit vos seins, au cours de la semaine dernière, dans quelle mesure avez-vous été satisfaite ou insatisfaite:
How normal you feel in your clothes?	Dans quelle mesure vous sentez-vous normal dans vos vêtements?	4	“Dans quelle mesure” is an unnecessary phrase at the start of several question items.	Vous sentez-vous normale dans vos vêtements?
How your lumpectomy breast sits/hangs?	Comment votre sein tombe-t-il après la tumorectomie?	4	“Tombe” translates to “falls” as opposed to “sits/hangs”	Comment votre sein pend ou se soutien depuis la lumpectomie?
**“Adverse Effects of Radiation (Postoperative)” scale**				
Your radiated breast skin looking different (e.g., too dark or too light)?	La difference de votre la peau sein irradié (ex: trop foncée ou trop pâle)?	4	“Difference” is missing an accent and should be “différence.”	La différence de votre la peau sein irradié (ex: trop foncée ou trop pâle)
Your radiated breast skin feeling sore (sensitive) when touched (e.g., changes in water temperature when you bathe/shower)?	La peau de votre sein sensible au toucher (par exemple, un changement de la température de l’eau pendant votre douche/bain)?	2	“Feeling sore” is absent from the translated version.	No change made.

Note: Only items with feedback from three or more patients were revised in the final Canadian French version

## Discussion

This study reports the linguistic and cultural adaptation of two BREAST-Q Breast Conserving Therapy module (version 2.0) scales *—* “Satisfaction with Breasts (Postoperative)” and “Adverse Effects of Radiation (Postoperative)”*—*into Canadian French. The findings demonstrate the value of a translation process involving native speakers and patient partners for producing PROMs that are both conceptually equivalent to the source material and culturally relevant to the target population. In an international trial context, where participant populations represent diverse linguistic and cultural backgrounds, the availability of translated study materials is important for ensuring accurate measurement of patient-reported outcomes, enabling equitable participation, and supporting robust cross-country comparisons [10, 12]. Within-country cultural and regional heterogeneity may also influence how participants’ interpret and respond to PROMs, highlighting the value of localized adaptations to enhance accessibility.

The cognitive debriefing interviews were a critical step in the translation process. Participants suggested refinements for eight terms or phrases, of which five were incorporated into the final version of the scales. Consistent with other translations of the BREAST-Q, patient partners in our study refined specific concepts to ensure conceptual equivalence and patient-centered clarity [9, 13, 14]. Within our study, patient feedback primarily concerned terminology and nuance; for example, “tombe” was replaced with “pend ou se soutient” to more accurately describe postoperative breast changes, and “lumpectomie” was preferred over “tumorectomie” to reflect terminology more familiar to patients. The latter choice is consistent with usage in patient education materials and published literature, underscoring how patient partners contribute valuable, experience-based perspectives that strengthen the cultural relevance of translated instruments.

This translation holds particular significance in the Canadian context, where Canadian French is an official language spoken in the provinces of Quebec and New Brunswick, and by sizable Francophone communities in other provinces such as Manitoba and Ontario [15]. In Quebec alone, 8,587 women were diagnosed with breast cancer in 2023 [16]; however, despite this substantial patient population, few validated PROMs exist in Canadian French for breast cancer care, limiting the ability to systematically capture patient experiences in their preferred language. A study translating the Patient-Reported Outcome Measurement Information System (PROMIS) item banks into French demonstrated that Canadian French possesses distinct lexical, semantic, and pragmatic nuances compared to other French varieties [17]. Translations of PROMs that do not account for these differences risk reduced comprehension and response accuracy among Canadian French study populations [12, 17, 18].

Language barriers are well-documented contributors to lower participation rates in cancer clinical trials [19], and the absence of validated measures in a patient’s primary language can lead to systematic exclusions of certain patient groups or misrepresentation of their experiences. [20–22]. Ensuring language-appropriate measures may enable patients to accurately convey their experiences and support greater patient-centred care [23, 24]. A notable limitation of this study is the small sample size of participants involved in the cognitive debriefing interviews. In addition, this study included the translation of two BCT scales; however, other scales are being translated into additional languages through ongoing international efforts [25]. Future research can focus on evaluating the psychometric properties of these scales within larger Canadian French-speaking populations across the country to ensure broader generalizability and validity.

## Conclusion

The Canadian French translation of the BREAST-Q BCT module enables breast cancer patients who have undergone BCT to accurately self-report outcomes. Involvement of patient partners was essential in producing translations that are both comprehensible and contextually appropriate, drawing on their lived treatment experiences. These translations represent an important step toward enhancing accessibility and representation in clinical trials.

## Data Availability

No datasets were generated or analysed during the current study.
